# CD34 and CD117 Stemness of Lineage-Negative Cells Reverses Memory Loss Induced by Amyloid Beta in Mouse Model

**DOI:** 10.3389/fnbeh.2018.00222

**Published:** 2018-11-01

**Authors:** Parul Bali, Sridhar Bammidi, Avijit Banik, Bimla Nehru, Akshay Anand

**Affiliations:** ^1^Department of Biophysics, Panjab University, Chandigarh, India; ^2^Neuroscience Research Lab, Department of Neurology, Post Graduate Institute of Medical Education and Research, Chandigarh, India; ^3^Department of Pharmacology, Rollins Research Center, Emory University School of Medicine, Atlanta, GA, United States

**Keywords:** memory loss, alzheimer disease, amyloid beta-peptides, CD117, CD34, stemness

## Abstract

A majority of the neurodegenerative disorders including Alzheimer's disease are untreatable and occur primarily due to aging and rapidly changing lifestyles. The rodent Alzheimer's disease models are critical for investigating the underlying disease pathology and screening of novel therapeutic targets in preclinical settings. We aimed to characterize the stemness properties of human umbilical cord blood (hUCB) derived lineage-negative (Lin−) stem cells based on CD34 and CD117 expression as well as surface morphology using flow cytometry and scanning electron microscopy, respectively. The efficacy of the stem cells was tested by its capacity to rescue the injury caused by intrahippocampal delivery of varying doses of amyloid beta. The hUCB Lin− stem cells reversed memory loss due to Aβ42-induced injury more effectively at micromolar concentration, and not picomolar concentration. More studies are required to delineate the underlying molecular events associated with hUCB Lin− stem cells.

## Introduction

Neurodegenerative disorders that have affected millions of people worldwide are untreatable and worsen with age. This includes Parkinson disease, ataxia, amyotrophic lateral sclerosis, glaucoma, Lewy body disease, microvascular brain injury, Alzheimer's disease (AD), and other dementias. Dementia has affected almost 47 million individuals at the age of 65 years and older. This number is projected to be more than 131 million in 2050 (Prince et al., 2016).

The drugs and therapies available at present provide only symptomatic relief without alleviating or halting the disease progression. Most of the drugs that have been successful in preclinical studies have failed during clinical trials (Banik et al., 2015a). Eli Lilly developed a drug solanezumab, targeting amyloid with an aim to delay the progression of AD and then subjected it to clinical trials, which eventually were terminated prematurely (Honig et al., 2018). There was repeated failure of clinical trials in AD; this failure calls for reinvigorated effort to discover effective biotherapeutic approaches (Saraf et al., 2011; Innes and Selfe, 2014; Eyre et al., 2016; Minhas et al., 2017). Simultaneously, there is an urgent need to test other alternative therapies.

Stem cell therapy for AD is one of the various alternative therapies for untreatable disorders. In the APP/PS1 transgenic mouse model, the transplantation of mesenchymal stem cells (MSCs) from human umbilical cord blood (hUCB) (Yang et al., 2013) and Wharton's jelly (Xie et al., 2016) has demonstrated the rescue of cognitive impairment by reducing amyloid deposits. However, none of these studies has characterized the cells used in transplantation.

The ultrastructure of embryonic stem cell-derived cardiomyocytes was characterized by visualizing under scanning electron microscopy (SEM) and transmission electron microscopy (TEM; Taha et al., 2012). Similarly, an overall protocol was standardized for TEM to visualize the ultrastructure details of adult MSCs as well (Miko et al., 2015). The analysis of CD34+ cells by TEM depicts the phenotypic immaturity in umbilical cord blood cells in comparison to cells derived from bone marrow (Deliliers et al., 2001). The flow cytometry analysis has shown that lineage-negative (Lin−) cells proliferative slowly but maintained long-term culture and are more primitive than CD133+ and mononucleated (MNC) populations (Forraz et al., 2004). However, further are required for stem cell characterization based on morphological and surface molecular marker assessments that are essential for any advancement in regenerative medicine.

The Lin− population has been identified as a group of cells participating in the reconstitution of formed elements in hematopoiesis. The Lin−Sca1+CD34+Flt+ cells have been shown to differentiate in lineages other than hematopoiesis. This aspect could be interesting in context to our study. The Lin− population comprises cells responsible for the formation of vascular endothelial cells (Asahara et al., 1997). The endothelial progenitor cells in turn can initiate a number of signaling cascades leading to vascularization (Kalka et al., 2000; Gill et al., 2001) in the sites of peripheral vasculature in ischemia (Asahara et al., 1997) or induced ocular injury (Grant et al., 2002). Reports revealed the complete prevention of vascular degeneration in the CNS derivatives (retina) in transgenic mouse models of neuronal degeneration (rd1 & rd10), which correlated to neuronal rescue (Otani et al., 2004). These findings show that Lin− stem cells act like endothelial precursor cells and stimulate neurotrophic responses in healing/to injury. Lin− stem cells, derived from the umbilical cord blood or bone marrow had earlier been used and their efficacy examined in several neurodegenerative mouse models (Banik et al., 2015b; Jindal et al., 2017; Minhas et al., 2017). These studies report the upregulation of the neurotrophic effect by brain derived neurotrophic factor (BDNF) and nerve growth factor (NGF) with the downregulation in GFAP (Minhas et al., 2017). It is suggested that Lin− stem cells might modulate neurotrophic factors when transplanted intravitreally near the site of retinal injury artificially induced by N-methyl-D-aspartate (NMDA). (Jindal et al., 2017). Even though our previous report also showed that hUCB-derived Lin− stem cells exert a therapeutic effect in a dose- and time-dependent manner (Banik et al., 2015b), the shape and size of the transplanted cells were not analyzed or characterized adequately.

The hUCB is an underutilized source of stem cells. Cell-based therapies hold an alternative yet untested promise for AD treatment and there are several cord blood banks worldwide promising such therapies to their clients; hence there is a pressing need to investigate the therapeutic potential of hUCB-derived stem cells. The cord blood banks preserve stem cells from hUCB, for its future use in several untreatable neurological disorders. This study was initiated to characterize the stemness of Lin− stem cells based on the surface marker CD34 and CD117 expression and its comparison with MNCs and lineage-positive (Lin+) cells. The 3D visualization, surface morphology, and size of all three cell types were analyzed using SEM. Further, we developed an animal model of memory loss induced by a higher dose (1 μM) of amyloid Beta-42 (Aβ42) and used this model to analyze the therapeutic efficacy of Lin− stem cells derived from hUCB against a higher dose of Aβ insult. This study aimed to increase the toxic effect in the brain, more precisely in the hippocampus, through a higher dose of 1 μM of Aβ42 compared to the dose of 800 pM used in our previous study. It was decided that this would test a better window of therapeutic action of Lin− stem cells in the injured brain. It was hypothesized that the more exacerbated brain pathology could exert an improved therapeutic effect of these naive stem cells to alter disease pathology. Memory loss in mice was induced by injecting an aggregated form of Aβ42 in their hippocampus. Their memory was analyzed by the Morris water maze (MWM) and passive avoidance tests. Significant impairment was observed in the spatial memory. Purified and enriched hUCB Lin− stem cells were also transplanted into the injury-induced hippocampal region of the mouse. The mouse group with the transplanted Lin− stem cells showed a reversal of memory loss caused by Aβ42. When compared to the earlier findings with a dose of 800 pM Aβ42, an improving effect on memory was observed at the same dose of stem cells transplanted but at a 1 μM dose of Aβ42. At present, it is not clear how a higher dose of Aβ42 could play a role in exerting a better effect of Lin− stem cells in improving the memory in the mice. In future studies, the aim is to focus on deciphering the putative molecular mechanism mediating the therapeutic effect of hUCB Lin− stem cells in the reversal of cognitive impairment.

## Materials and methods

All experiments were conducted in a good laboratory practice-compliant lab. Standard operating protocols were prepared and used for the study, following the approval by the quality assurance personnel and the study director. All the data entries and protocols were recorded in data recording sheets and the experimental values or results were verified by independent personnel, duly documented in a verifiable and auditable format.

### Study plan

#### Preparation of amyloid β aggregates

An amount of 0.1 mg of Aβ42 with empirical formula C_203_H_311_N_55_O_60_S and molecular weight 4514.04 (Sigma-Aldrich) was procured. This was dissolved in 100 μl of 1× phosphate buffer saline (PBS) with pH adjusted to 7.4. In accordance with previously published protocols for Aβ42 oligomerization, the peptide was kept in incubation at 37°C for 4 days and 4°C for 6 h (Ahmed et al., 2010).

#### Isolation of lin− stem cells from hUCB

HUCB was collected after the delivery of newborns from mothers aged 20–35 years (≥28 weeks gestation). The samples were collected in accordance with the ethical guidelines approved by the Institutional Committee on Stem Cell Research and Therapy (ICSCRT; Approval no. IC-SCRT/03/DTM-2979). Samples were collected after obtaining informed consents from the participants. The UCB was taken from placental and umbilical cord blood vessels using 21-gauge sterile needles in an EDTA (anticoagulant) containing vial. The mononucleated cell (MNC) population was isolated by the Ficoll density gradient method. This population of cells was subjected to magnetic assisted cell sorter (MACS) using a cocktail of antibodies for the Lin+ cell surface marker tagged with magnetic beads. The Lin− population was enriched via negative selection when MNCs were incubated with the Lin+ antibody cocktail and allowed to pass through a magnetic column. The Lin+ population was collected after removing the column from the magnetic field.

### Characterization of stem cells isolated from HUCB

#### SEM analysis

The UCB-derived MNCs, Lin+ cells, and Lin− cells were plated separately on 12 mm circular cover slips in a 24-well culture dish and incubated at 37°C for 1 h. Samples were immersed in 3% glutaraldehyde buffered with 0.1 M Sorensen's buffer at 0–4°C for 48–72 h. This was then rinsed thrice with 1× Sorensen's buffer for 30 min each. The cover slips containing the cells were then subjected to serial dehydration. The coverslips were dipped into 30% → 50% → 70% → 80% → 90% → 95% → 100% → 100% concentrations of ethanol for 10 min each. The cover slips were dried and mounted on the stubs and the samples coated with platinum. Platinum coating was done in an Auto Fine Coater (JEC3000FC, Jeol, Japan) by platinum sputtering. A current of 20 mA was used with an exposure time of 40 s, under vacuum. The samples were then visualized by using a scanning electron microscope (JSM-IT300, Jeol, Japan). They were scanned using a secondary electron detector at a voltage of 9.0 kV and a probe current of 40.0 A, under high vacuum. Images were acquired at 1000×, 2000×, and 5000×.

#### Flow cytometric analysis

A flow cytometric analysis was also carried out to determine the percentage expression of CD45 (nucleated cell marker), CD34, CD117 (stem cell markers), and CD271 (mesenchymal marker) in these cells isolated from hUCB. About one million cells from each population were suspended in 100 μl FACS buffer (PBS-BSA-Azide solution) and incubated with Fc blocker (20 μl for up to 10^7^ cells) (Miltenyi Biotech, USA) for 30 min at room temperature (RT). Then fluorochrome-conjugated antibodies (BD Pharmingen, USA) were added in the tubes as per the requirement and incubated for 1 h at RT. CD45 markers were tagged to FITC, CD34 were tagged to PE, CD117 were tagged to APC, and CD271 were tagged to Cy3 or PerCP fluorophore conjugates. Finally, all the tubes were washed twice with FACS buffer and resuspended in 300 μl buffer and analyzed in FACS Calibur (BD Bioscience, USA) within 2–6 h of processing.

#### Animals

Six- to eight-weeks-old inbred Swiss albino mice were used after approval from the Institutional Animal Ethical Committee (IAEC-473). Animals were maintained in a 12 h light–dark cycle (LD 12:12). These were fed on a standard diet and had free access to drinking water. Mice were sacrificed using an overdose of anesthesia i.e. xylazine and ketamine cocktail. Brains were immediately isolated and stored at −80°C till further use.

#### Intrahippocampal delivery of Aβ42 and lin− stem cells using stereotaxis

Six- to eight-weeks-old mice were injected with Aβ peptide using stereotaxis. Mice were anesthetized by an intraperitoneal injection of xylazine (10 mg/kg)–ketamine (100 mg/kg) cocktail. The mouse was then positioned on the stereotaxis apparatus in the prone position, with their ears and front teeth were fixed to prevent any head movement. The skull was exposed by giving an incision on the scalp in the median axis. In the exposed skull, Bregma zero was taken as the reference point and a microsyringe needle was moved to specific Bregma coordinates: anteroposterior (AP) +2 mm, mediolateral (ML) ±2 mm, and dorsoventral (DV) −2.5 mm for specific delivery at the dentate gyrus in the hippocampus region, as previously standardized in the lab.

Craniotomies for the bilateral injection, by exposing the skull and injection points, were marked using a 26G needle according to the Bregma scale, following the stereotaxic coordinates (Paxinos and Franklin, 2004). A 1 μM concentration of aggregated Aβ solution, in 5 μl of PBS, was injected at a rate of 1–2 μl/min using a rate-controlled microinjector. After the solution was delivered in the hippocampus, the needle of the microsyringe was kept unmoved for 5 min for proper diffusion of the solution and then slowly removed from the brain by unscrewing the arm of the injection. In the vehicle-treated group, PBS was injected bilaterally and a needle was inserted in the sham surgery group without any Aβ42/vehicle delivery. Similarly, either 50,000 hUCB Lin− stem cells suspended in 1× PBS or 1× PBS alone as vehicle were transplanted at the same site of injury after 21 days of Aβ42 injection using stereotaxic apparatus.

### Behavioral analysis

#### Evaluation of spatial memory by morris water maze (MWM) experiment

MWM was performed to evaluate the spatial memory of the mice subjected to various experimental conditions, i.e., Aβ42-injected (injury) group, vehicle control, sham control, and stem cell-transplanted group at day 10 post-transplantation. Before subjecting the mice to MWM, the mice were screened for their swimming ability and motor functioning. On day 0, mice were allowed to swim freely for 2 min to examine their swimming ability and subjected to rotarod screening and excluded from the study if found to have irregular muscle coordination. The mice with normal behavioral pattern and vision were included for further experimentation in MWM. The basic MWM protocol for the navigation task included a circular pool where visual cues were placed on the walls and pool side. The tank was divided into four artificial equal quadrants—T1, T2, T3, T4—and a hidden platform was placed in compartment T1 submerged 1 cm below the water surface. The protocol of seven days was designed and included 6 days acquisition and 7th day retrieval, each day consisting of 4 trials and each trial lasting 120 s. The entire experiment was video-tracked using automated Anymaze software connected with a webcam, which was mounted to obtain an aerial view of the pool. The water temperature was maintained at 21°C (Vorhees and Williams, 2006; Weitzner et al., 2015) and colored black to provide a contrast of white Swiss albino mice so that Anymaze software can identify, distinguish, and track the animal against the background. Using the Anymaze software, the experiment was designed by marking the marginal area of the pool divided into the four quadrants and the hidden platform. In the protocol, events and trials were assigned e.g., entry to platform area, entry to each quadrant etc. The various parameters were analyzed, including escape latency time, mean speed, time spent in each quadrant, distance from each quadrant, and mean distance from the platform (search error). The escape latency time was compared in the different groups. Further, the swimming track plots from all the trials were recorded to analyze their index of learning.

#### Passive avoidance

This is a fear-aggravated test in which mice subjected to various experimental conditions are evaluated for learning behavior. The equipment is made up of one lit compartment and one dark compartment. The 3-day experiment was set up and each trial lasted maximum 5 min. On the first day, the mice were kept in a lit chamber and allowed to move freely. After 24 h, in the acquisition/condition phase, mice were kept in the lit chamber. When a mouse moved to the dark chamber, it received a mild foot shock of 20 mA. On the test day, the latency time, i.e. the time taken by the mice for crossing the gate to avoid the stimulus, was calculated and noted; this latency time is associated with memory and learning. The groups were analyzed and compared for the results to analyze the learning-associated memory.

#### Congo red staining

The Aβ42 aggregates were identified using Congo red staining of brain cryosections of the hippocampal region. The cryosections were fixed using histochoice and then hydrated with 90, 70, and 50% ethanol, followed by washing in distilled water. The slides were stained with 1% alcoholic Congo red solution for about 30 min at RT and the nuclei were counterstained with hematoxylin. Excess staining were removed by immersing the slides in 70% alcohol for a few seconds and then cleared in xylene for 30 min before mounting it with a fluorosave mounting medium (Calbiochem, USA).

#### Immunohistochemistry

To further confirm the Aβ42 aggregates, immunohistochemistry was performed. The Aβ42 primary antibody (Elabscience) was used at 1:100 dilution and incubated overnight at 4°C after serum blocking. The TRITC donkey antirabbit secondary antibody was used at 1:200 dilutions. Washing was done using 1× PBS and counterstained with DAPI. The sections were analyzed using a 532 nm laser line for excitation in confocal microscopy (Olympus FV1000) and the images were merged using its software.

#### Statistics

All results were analyzed as mean ± SEM in Microsoft Excel. The data were arranged and statistically analyzed using SPSS software version 16.0. In MWM, repeated-measure ANOVA was used for repetitive observation on acquisition days and retrieval trial. Further, a *post hoc* analysis was carried out using least significant difference (LSD). In the passive avoidance test, an independent *t*-test was applied. The values were considered statistically significant if *p* ≤ 0.05 in the results.

## Results

### Standardization of bregma coordinates for hippocampal injection

**Figure 1:**
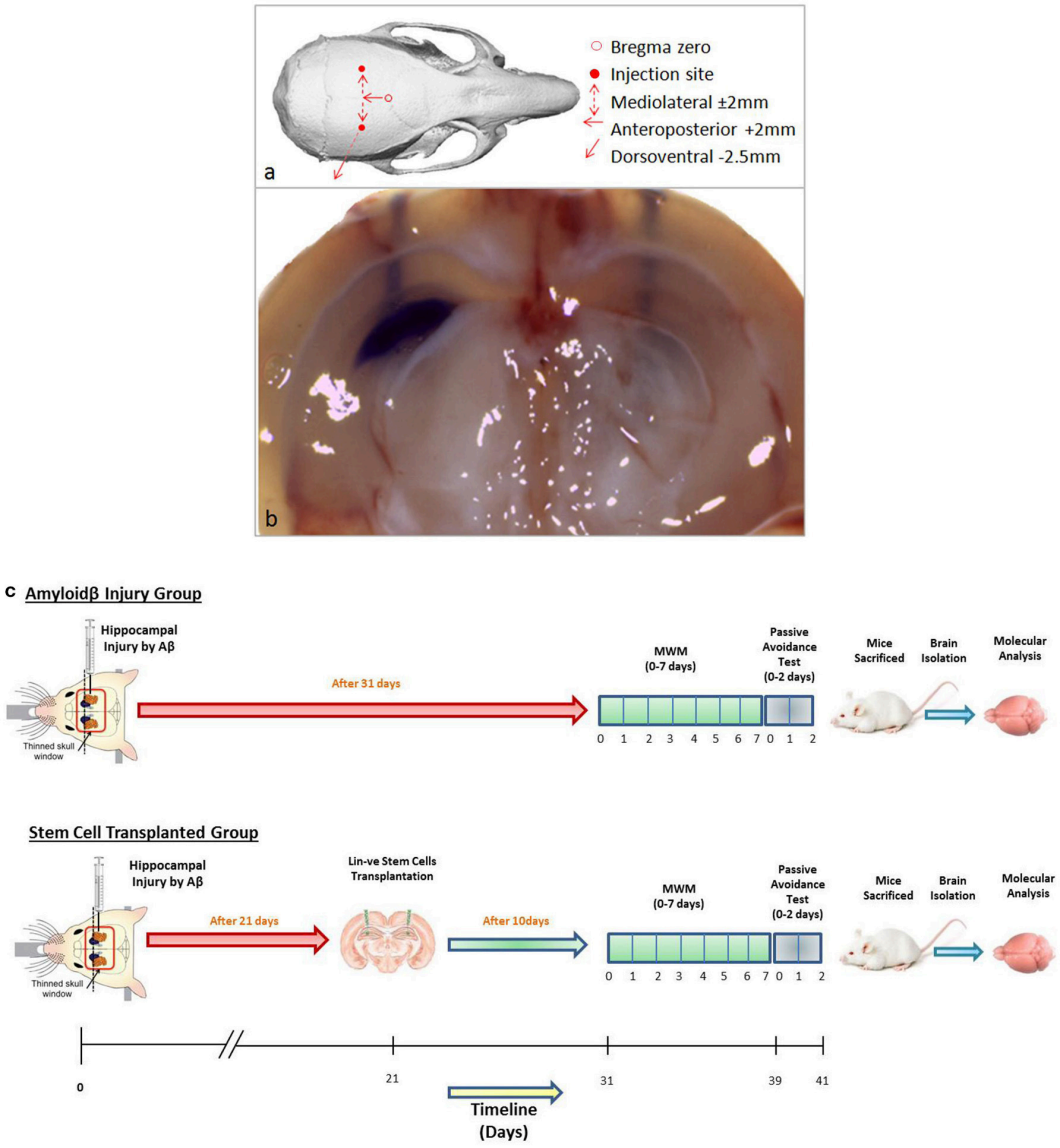
**(a)** Schematic representation of mouse skull bones showing Bregma zero point and site of injection for hippocampal delivery. **(b)** The gross coronal section of mouse brain shows the injected 2 μl of crystal violet dye diffused throughout the hippocampal area with a needle track on the right hemisphere. In the left hemisphere, a needle was inserted without injecting crystal violet. **(c)** The schematic of the *in vivo* study design of the Aβ injury group and the stem cell-transplanted group.

Memory loss was induced in 6 to 8-weeks-old Swiss albino mice using intrahippocampal Aβ42 injection by stereotaxic surgery. The schematic represents the skull sutures in the exposed mice brain and the Bregma zero point, from where the axis for hippocampal region was located (Figure 1a). For intrahippocampal delivery, bregma coordinates of the skull were standardized by injecting crystal violet dye at anteroposterior axis +2 mm, mediolateral axis −/+ 2 mm, and dorsoventral axis −2.5 mm. The crystal violet dye dispersed throughout the hippocampus with a prominent needle track in the right hemisphere, shown in the coronal section visualized under a dissecting microscope, and only a needle track in the left hemisphere where a needle was inserted without injecting the dye (Figure 1b). Further, these coordinates were used for Aβ42 injection and hUCB Lin− stem cell transplantation.

### SEM characterization of stem cells isolated from hUCB

**Figure 2:**
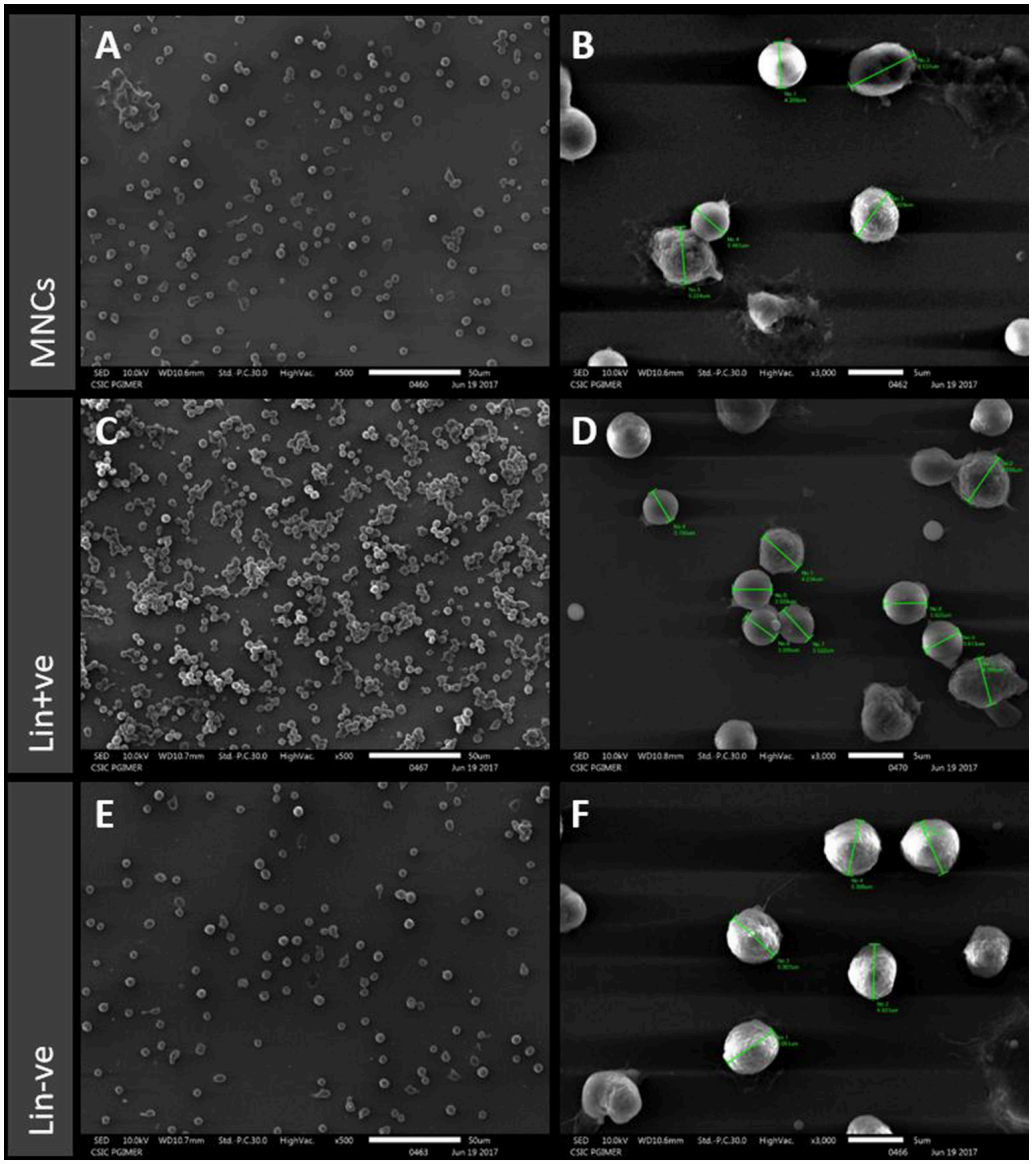
Scanning electron microscopy (SEM) images of MNCs **(A,B)**, Lin+ **(C,D)** and Lin− **(E,F)** from hUCB for morphological characterization. MNCs show heterogeneous populations with variation in shape, size, and structure. The Lin+ cells show similar heterogeneous populations and clusters around even-sized microbeads whereas the Lin− cells show homogenous population and have the same shape, size, and structure.

SEM analysis revealed the morphology and size of all the three cell types isolated from hUCB (Figure 2). MNCs show heterogeneous populations of immature RBCs and varying lymphocytes. They also show variation in shape, size, and structure. The MNC population was found to be of varying size ranging from 3 to 6 μM in diameter (Figures 2A,B). Lin+ cells were found to be in clusters with even-sized microbeads (Figure 2C) and they also showed heterogeneous populations with varying size similar to MNCs (Figure 2D). Lin− cells showed homogenous population with the same shape, size, and structure. These cells were 5 μM in diameter and uniformly distributed (Figures 2E,F). There were no magnetic beads found to be tagged to these cells, confirming their purification by negative selection in a magnetic field.

### Flow cytometric analysis of stem cells isolated from hUCB

**Figure 3:**
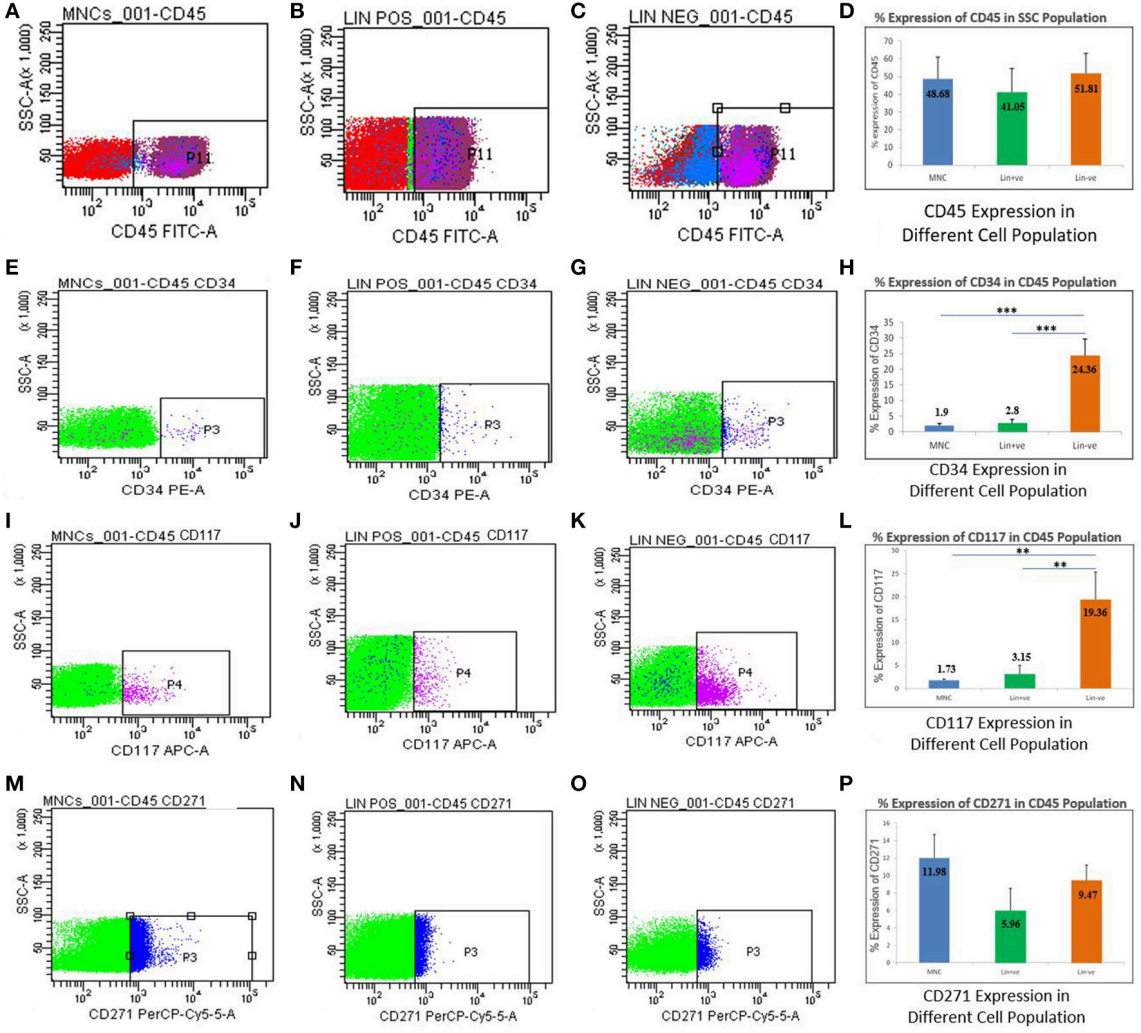
Flowcytometric analysis for MNCs, Lin+ cell, and Lin− cell populations isolated from hUCB. Percentage expression of CD45 showing representative dot plots on three different populations in hUCB **(A–C)** and their average expression shown in a bar graph. **(D)** Representative dot plots showing the expression of CD34 out of CD45 positive cells **(E–G)**. Graph showing the expression of CD34 higher in Lin− cells as compared to MNCs and Lin+ cells **(H)**. Representative dot plots showing the expression of CD117 out of CD45 positive cells **(I–K)**. Graph showing the expression of CD117 higher in Lin− cells as compared to MNCs and Lin+ cells **(L)**. Representative dot plots showing the expression of CD271 out of CD45 positive cells **(M–O)**. Graph showing the expression of CD271 is not significantly different in MNCs,Lin+ and/or Lin− cells **(P)**. *N* = 13, **p ≤ 0.01, ***p ≤ 0.001.

All the three cell types isolated from hUCB were analyzed in a flow cytometer for the presence of nucleated marker (CD45), stem cell markers (CD34, CD117), and mesenchymal markers (CD271; Figure 3). When the side scatter population was gated against the CD45-FITC channel, the proportional expression of CD45 was comparable to MNCs (48.68%), Lin+ (41.05%), and Lin− (51.81%; Figures 3A–D). Further, CD34 and CD117 percentage expression was gated among the CD45 positive cells from all the cell types. In both the cases, CD34 and CD117 stem cell expression was found to significantly increase in Lin− cells as compared to MNCs and Lin+ cell types. CD34 was highly expressed (24.36%) in Lin− cells, while in MNCs (1.9%) and Lin+ cells (2.8%), reduced expression was noted (Figures 3E–H). Similarly, CD117 expression was also found to be significantly high in Lin− cells (19.36%) as compared to MNCs (1.73%) and Lin+ cells (3.15%; Figures 3I–L), suggesting that Lin− cells are an enriched population of stem cells. We also examined the presence of CD271, a mesenchymal marker in the stem cell population. The data showed reduced percentage of CD271 (5–12%), which was comparable among all the cell types (Figures 3M–P). This shows that Lin− enrichment comprised the nascent hematopoietic stem cell population. However, no other lineages such as mesenchymal lineage were observed.

### Brain sections reveal Aβ42 deposition and hUCB lin− stem cells upon transplantation

**Figure 4:**
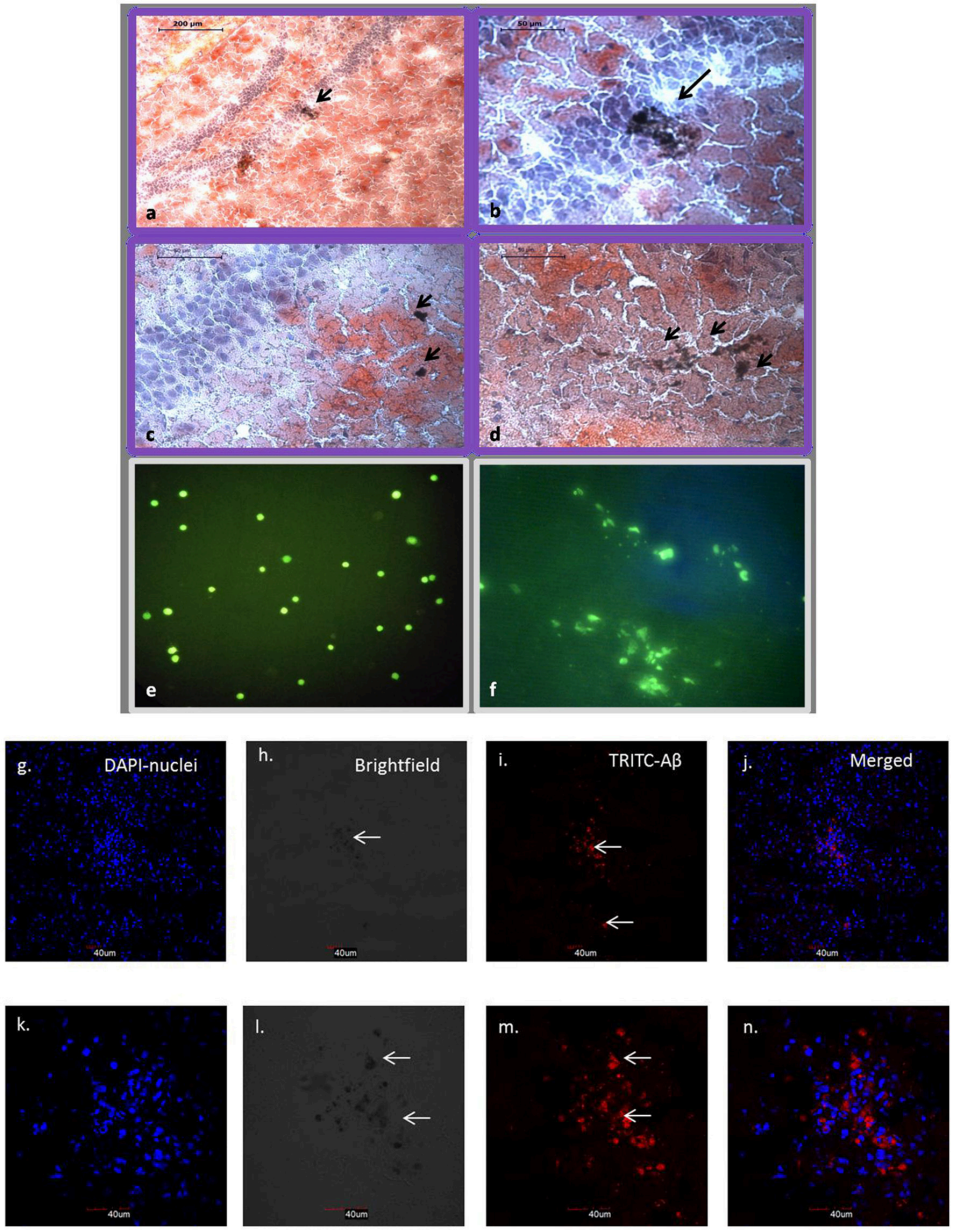
Brain sections with Congo red staining show dark brown Aβ42 aggregates in and around hippocampal region **(a–d)**. **(a)** 10× view of hippocampal region with arrow showing Aβ42 deposition. **(b–d)** 40× view with arrows showing Aβ42 deposition in the dentate gyrus region. CFDA labeled hUCB Lin− stem cells before and after transplantation (*N* = 3; **e,f**). **(e)** Confocal image shows CFDA positive (green) MACS sorted hUCB Lin− stem cells before transplantation. **(f)** Transplanted cells were identified in brain sections upon 10 days post transplantation under the confocal microscope (Figure 2B). **(g–n)** Further, Aβ42 aggregates were confirmed by immunohistochemistry at 20× **(g–j)** and 60× **(k–n)** magnification using confocal microscopy (*N* = 3). Aβ42 aggregates were seen in TRITC filter at 532, which gives red fluorescence tagged to secondary antibody **(i,m)**; nuclei were counter stained with DAPI (blue; **g,k**) and aggregates were also seen as dark spots in bright field **(h,i)**.

Stereotaxic surgery was performed to deliver 1 μM concentration-aggregated oligomers of Aβ42. To identify the amyloid-aggregated brain, sections were stained with alcoholic Congo red (Lorenzo and Yankner, 1994). Congo red binds to the Aβ42 aggregates and imparts dark red/brown color. Stains were identified near the dentate gyrus region of the hippocampus, confirming the deposition of injected Aβ42 in the mouse brain (Figures 4a–d). Further, immunohistochemistry confirms the presence of Aβ42 aggregates that were analyzed using confocal microscopy (Figures 4g–n). MACS-sorted hUCB Lin− stem cells were pre-labeled with CFDA dye and transplanted at the site of injury. Transplanted cells were identified in brain sections upon 10 days post-transplantation under the confocal microscope (Figures 4e–f).

### Aβ42-induced memory deficits were reversed by hUCB lin− stem cells

**Figure 5:**
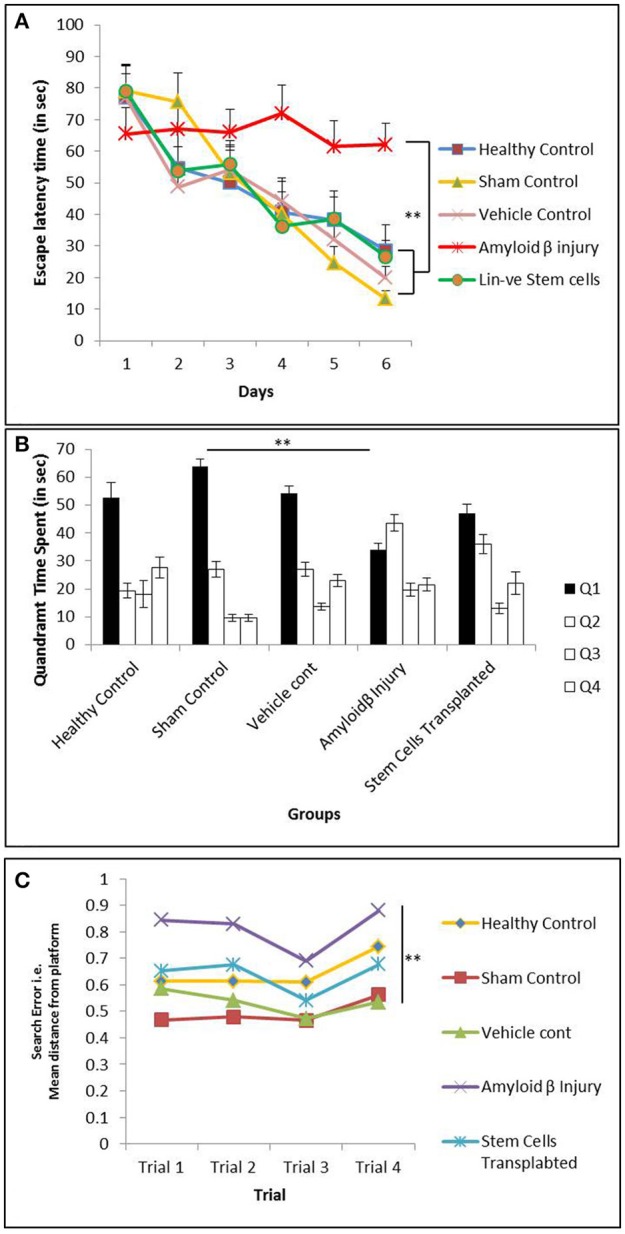
The MWM analysis showing memory impairment in mice injected with Aβ42 and further recovery after hUCB Lin− stem cells transplantation. **(A)** Graph depicts day-wise escape latency time (ELT) (s) during acquisition trials taken by mice in healthy control (*N* = 5), sham control (*N* = 7), vehicle control (*N* = 10), Aβ injury (*N* = 7), and Lin− stem cell-transplanted (*N* = 8) groups. Aβ42-injured mice showed significant memory loss by their never reducing ELT compared to healthy control, whereas hUCB Lin− transplanted mice with Aβ42 injury showed ameliorated memory with reducing ELT along the acquisition days 1–6. **(B)** Retrieval trials on day 7 showed quadrant time (s) spent by mice from different groups in the four Q1, Q2, Q3, and Q4 quadrants. Aβ42-injured mice could not spend maximum time in target quadrant (Q1) as that spent by the control mice, whereas after hUCB Lin− transplantation mice recovered their retrieval memory depicted by their maximum time in Q1 searching for the hidden platform. **(C)** The search error during retrieval trials (day 7) showing the mean distance from the hidden platform was found to be significantly increased in Aβ42-injured mice while it was significantly decreased in Lin− transplanted mice suggesting recovery in memory. Data were analyzed using SPSS repetitive measure ANOVA test followed by LSD *post hoc* analysis (**p ≤ 0.01).

After Aβ injection, the mice were tested for memory loss with the Morris water maze (MWM) experiment. Spatial memory was further tested by subjecting the mice to the MWM on the 10th day post-transplantation of hUCB Lin− stem cells (Figure 1c). The increased escape latency time (ELT) indicated the significant spatial memory loss in mice treated with Aβ42. The mice transplanted with hUCB Lin− stem cells showed day-wise decrease in escape latency comparable to the mice in the healthy, vehicle, and sham control groups. The HUCB Lin− stem cell transplantation group showed a significant improvement in special memory as compared to the Aβ42 injury group (Figure 5A). The quadrant time spent by mice was calculated in each quadrant of the MWM tank on retrieval day (day 7). In Figure 5B, the amyloid injury group mice showed less time spent in target quadrant Q1 as compared to healthy, vehicle, and sham control groups, whereas more time was spent by mice transplanted with hUCB Lin− cells. In Figure 5C, the mean distance from the platform traveled on retrieval day (day 7) in four consecutive trials by the mice was calculated and plotted. The results depict that increased mean distance (from the platform) was traveled by amyloid-induced injury mice as compared to mice in control groups, and this distance was again reduced significantly in the stem cell-transplanted group in comparison to the injury group.

### Swimming track plots confirm loss of memory in Aβ42-injured mice and recovery in memory after hUCB lin− stem cell transplantation

**Figure 6:**
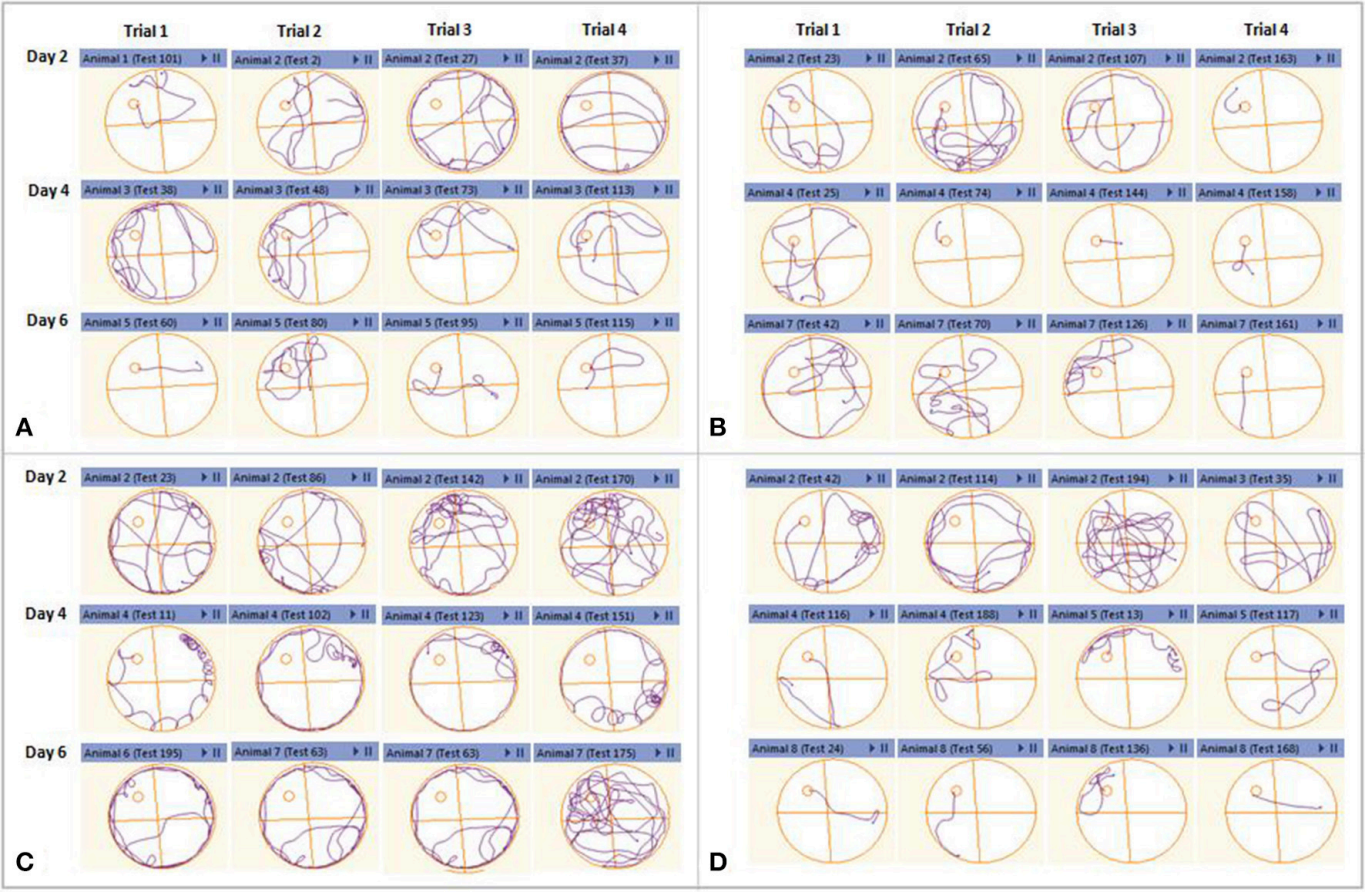
Representative swimming track plots from acquisition day 2, 4, and 6 of different groups as an index of learning. Each day had four consecutive trials. **(A)** Healthy control and sham control. **(B)** Mice showed reduction in swimming path to reach the hidden platform along the acquisition days. **(C)** Aβ42-injured mice could not reduce their swimming path as the acquisition days progress. **(D)** After hUCB Lin− stem cell transplantation mice showed striking reduction in their swimming path to reach the platform along the days of acquisitions as marked in the control groups.

Anymaze software-assisted swim track plots for all the trials were recorded for analysis using an automated camera. The track plots observed on day two, four, and six of the acquisition trials were evaluated for healthy, sham control, amyloid injury, and stem cell-transplanted groups. The healthy and sham control mice were found to have reduced swimming path with acquisition days. The Aβ42-injured mice were found to be swimming toward the edges of the MWM tank instead of moving near the platform, suggesting their memory impairment. These Aβ42-injured mice after hUCB Lin− stem cell transplantation showed striking reduction in their swimming path to reach the platform along the days of acquisitions as marked in the control groups (Figure 6).

### Passive avoidance analysis showed significant improvement of fear conditioning memory after hUCB lin− stem cell transplantation

**Figure 7:**
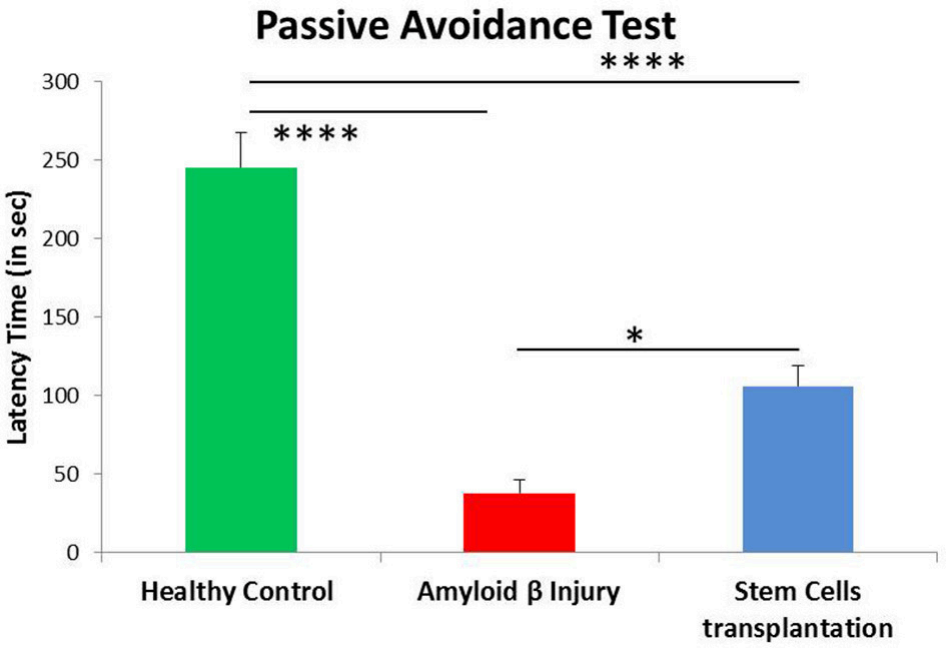
Passive avoidance analysis showed significant improvement of fear conditioning memory after hUCB Lin− stem cell transplantation. Aβ-injured mice (*N* = 8) showed significant reduction in latency time compared to healthy control mice (*N* = 7; ****p < 0.0001), whereas latency time was significantly increased after Lin− stem cell transplantation (*N* = 8; *p < 0.022). Data were analyzed using SPSS one-way ANOVA.

To further confirm the memory and learning in different groups, mice were subjected to another behavioral assay i.e., passive avoidance. The time taken by mice to avoid an aversive stimulus (i.e., electric shock) is proportional to the index of learning and memory. Memory performance is positively associated with the time taken by mice to move from the lit compartment to the dark compartment; more latency denotes a strong recollection of fear conditioning. The results showed a significant difference between control and Aβ42-injured mice (*p* < 0.001), with reduced latency time in Aβ42-injured mice. When these mice were transplanted with hUCB Lin− stem cells, the latency time significantly increased in passive avoidance (*p* < 0.001). This experiment further confirms memory loss in amyloid injury group and recovery by stem cell transplantation (Figure 7).

### Higher dose of Aβ-42 (1 μM) was found to be more effective than 800 pM in exerting the therapeutic effect of lin− stem cells

A previous study had shown that a lower dose (800 pM) of Aβ42 could exert a cognitive deficit in mice, and a dose- and time-dependent improvement in memory upon hUCB Lin− stem cell transplantation (Banik et al., 2015b). This study aimed to test the efficacy of Lin− stem cells on the impact of a higher dose (1 μM) of Aβ insult. In MWM analysis, although the acquisition and retrieval trials recorded a significantly higher ELT and quadrant time, respectively for groups treated with 800 pM compared to 1 μM Aβ, but interestingly, the stem cell-transplanted groups showed significant cognitive improvement with 1 μM in Aβ-injured mice compared to the mice that received the 800 pM dose, as evident from the recorded ELT, quadrant time, and search error analysis (Supplementary Figure 1). In acquisition trials, the difference in ELT between Aβ-1 μM and Aβ-1 μM+Lin− SC was found to be significantly higher (*p* < 0.001) than the difference between Aβ-800 pM and Aβ-800 pM+Lin− SC (*p* < 0.05). In retrieval trials, there was a significant difference between Aβ-1 μM and Aβ-1 μM+Lin− SC groups in quadrant time (*p* < 0.05) and search error analysis (*p* < 0.05) while these differences were found to be non-significant between Aβ-800 pM and Aβ-800 pM+Lin− SC groups. These findings strongly suggest that a higher dose (1 μM) of Aβ could exert an increasing therapeutic effect of Lin− stem cells in this cognitive impairment model.

## Discussion

Various animal models are in use for understanding memory loss and dementia and to validate therapies. We created Aβ-induced injury in a mice model of cognitive impairment as it is one of the most common hallmarks of AD. We first validated the effect of Aβ42 on memory loss in the mouse model using the Morris water maze and passive avoidance behavioral tests. The structural analysis of Aβ42 has shown that it exists in monomeric, oligomeric, and fibrillary forms (Hepler et al., 2006). The monomeric form is neuroprotective in nature (Giuffrida et al., 2009), whereas oligomeric forms exert neurotoxicity in the brain by impairing synaptic plasticity (Cleary et al., 2005). The oligomeric preparation was made as per the published protocol (Ahmed et al., 2010). It was then administered stereotactically via intrahippocampal delivery, and the memory loss was evaluated by the behavioral experiments mentioned above. The parameters were analyzed using escape latency time, quadrant time, and the mean distance from the platform, which indicated the memory loss induced by Aβ42. The video track plots further confirmed behavioral alteration in the swimming pattern in the treated mice. The results suggest that the oligomeric form of Aβ42 induces memory loss at 31 days after intrahippocampal injection. As the current therapeutic treatments against Alzheimer's and dementia only provide symptomatic relief without alleviating disease pathology and have failed to show any therapeutic benefits in clinical trials (Doody et al., 2014), Lin− stem cells derived from hUCB were used to simulate the microenvironment in the proximity of artificially induced neurotoxic plaques so that their clearance could be examined (Tong et al., 2015; Wang et al., 2015). The bilateral transplantation of Lin− stem cells from hUCB in the intrahippocampal regions of Aβ-injured mice was carried out. SEM analysis revealed that Lin− cells possess homogenous morphology with similar shape, size, and structure. The absence of magnetic beads around these cells also confirmed that these are the enriched population of stem cells without any markers for Lin+ cells. Further, these cells also showed a significantly higher percentage expression of stem cell markers such as CD34 and CD117 compared to MNCs and Lin+ cell types. Mesenchymal marker, CD271 expression on these cells confirmed the homogeneity of the transplanted cells. These findings suggest that these cells are the naïve cells present in the hUCB population and might be capable of exerting therapeutic effects in the injury model.

Approximately 50,000 hUCB Lin− stem cells were purified and transplanted in Aβ42-injected mice. This showed reversal in spatial memory and working memory after 10 days of transplantation. We used a higher dose (1 μM) of Aβ42 than the previously used 800 pM (Banik et al., 2015b) to test whether Lin− stem cells are able to rescue the behavioral phenotype even at higher doses. We report that 1 μM of Aβ42 is not only a better dose of injury but also provides a better substrate for Lin− cells to significantly improve learning and memory when transplanted. Earlier findings have revealed that aggregated oligomeric forms of Aβ42 are the most neurotoxic in nature (Cleary et al., 2005; Ahmed et al., 2010), whereas a lower dose of Aβ, in nanomolar concentration, can exert a neuroprotective effect through its antioxidative effects evident from the CSF analyzed lipoproteins (Kontush et al., 2001). Aβ in the CNS exerts effects that range from amyloid angiopathy (Attems et al., 2010) to associated neurotoxicity in the AD brain. It was interesting to note that the higher concentration of Aβ (1 μM) displayed a lower decline in cognition as compared to a low concentration of Aβ (800 pM) in the “injury only” mice. Although a high dose of Aβ is expected to exert severe injury in the brain, the duration (21 days) after which the mice were subjected to spatial memory assessment could have provided sufficient time to mount a superior compensatory response as compared to injury at lower concentration at the same time point. It can, therefore, be concluded from the previous findings that severity, location, and time of analysis may impact the neurobehavioral outcomes in patients also. Consequently, this may influence the healing process in an injured brain (Anderson et al., 2011). Although we cannot ascribe the cause of increased cognitive impairment to 800 pM Aβ-injured mice, we speculate that the increased number of mast cells and the microglial cell recruitment at the site of insult after severe injury (1 μM of Aβ) may have more effectively ameliorated the recovery cascade after 3 weeks of injury as compared to the other dose. This could have led to a differential neurobehavioral outcome as compared to 800 pM Aβ-injured mice (Kempuraj et al., 2017).

The extensive literature on stem cell transplantation studies has focused on embryonic and Induced pluripotent stem cells (IPSCs), completely neglecting the potential of autologous transplantation of stem cells banked in commercial cord banks, although there are limited studies. The intravenous transplantation of MSCs shows the modulation of the inflammatory environment in the traumatic brain injury (TBI) animal model, mediated by the enhanced expression of anti-inflammatory cytokines and the reduction in proinflammatory cytokines (Zhang et al., 2013), appearing to indicate the potential of biotherapeutics over synthetic drug development strategies. The bilateral hippocampal transplantation of MSCs isolated from UCB in the APP/PS1 transgenic mice model has also resulted in the reduction of Aβ plaques by an increased expression of neprilysin (Kim et al., 2012), but this has not been followed up with additional studies. Similarly, UCB-MSCs have shown improvement in the cognition and reduction of Aβ in APP/PS1 double transgenic mice at 33 weeks 4 days analysis (Lee et al., 2012). In the amyloid-infused model, MSCs have been shown to increase hippocampal neurogenesis and differentiation of neuronal precursor cells (NPCs) by the Wnt signaling pathway when analyzed at 2 and 4 weeks post-transplantation (Oh et al., 2015). Similarly, the neural stem cell transplantation in the triple transgenic mice model, i.e., APP/PS1/tau, has shown BDNF-induced amelioration of spatial memory and increased synaptic plasticity (Blurton-Jones et al., 2009). The adipose-derived stem cells migrated to the brain by crossing the blood brain barrier when infused intravenously in the Tg2576 mice model of AD and subsequently ameliorated memory loss by upregulated expression of VEGF and IL10. Together, these studies provided the rationale for testing the effects of stem cell transplantation in the Aβ-injury mouse model.

Even though these preclinical studies have shown variable effects using different sources of stem cells, the current study points out that the stem cell effects are either borne out by their paracrine effects or mediated by inducing the proliferation of endogenous stem cells (Ryu et al., 2016). In other models of AD, stem cells have been shown to increase the expression of synaptic proteins and ameliorate the disease pathology, which was not under testing here. Other studies also show the immunomodulatory action of stem cells by changing the expression of anti-inflammatory and proinflammatory cytokines as well as microglia activation. These cells have been shown to translocate at the site of injury and show homing and differentiation in the niche. They also exhibit paracrine effects and trigger endogenous/exogenous healing responses. Our previous study identified transplanted cells even 60 days after transplantation in the mouse brain, but these cells were not found to be differentiated into any neuronal cell types (Banik et al., 2015b). Although the present study did not elucidate the biochemical or molecular pathway involved in the behavioral outcome, we speculate that this therapeutic effect of the intrahippocampal transplantation of stem cells could be mediated by the paracrine effects of neurotrophic factors, such as GDNF, CNTF, and BDNF rather than direct replacement of damaged neurons. The secretion of neurotrophic factors, especially BDNF, has been shown to result in increased CREB phosphorylation via the TrkB pathway in other systems (Guo et al., 2017). This might activate genes that regulate cognitive function, neurogenesis, cell proliferation, differentiation, cell migration, or synaptogenesis. Future mechanistic studies could be carried out by administering the inhibitors of BDNF and CREB to investigate how Lin− stem cells promote learning through the BDNF–CREB pathway, if at all.

## Ethics statement

The stem cells were used after approval by Institutional Committee on the Stem Cell Research and Therapy (ICSCRT; Approval no. IC-SCRT/03/DTM-2979). Swiss albino mice were used after approval from Institutional Animal Ethical Committee approval (IAEC-473).

## Author contributions

PB conducted all the experiments, data analysis, and writing. SB conducted work of stem cell characterization and data analysis. AB was involved in manuscript writing and data analysis. BN contributed to co-conceptualization of manuscript. AA was involved in the complete conceptualization and writing of the manuscript.

### Conflict of interest statement

The authors declare that the research was conducted in the absence of any commercial or financial relationships that could be construed as a potential conflict of interest.

## Supplementary material

Supplementary Figure 1 MWM analysis shows the difference between 800 pM and 1 μMAβ treated groups (*N* = 7) with their respective Lin− stem cell transplantation groups. Stem cell-transplanted groups showed significant improvement in cognition in 1 μM of Aβ-injured mice (*N* = 8) compared to 800 pM dose. **(A)** In acquisition trials, difference in ELT between Aβ-1 μM and Aβ-1 μM+Lin− SC was significantly higher (^**^*p* < 0.001) compared to difference between Aβ-800 pM (*N* = 5) and Aβ-800 pM+Lin− SC (*N* = 5; ^*^*p* < 0.05). **(B)** In retrieval trials, the quadrant time was significantly improved in Aβ-1 μM+Lin− SC groups compared to Aβ-1 μM (^*^*p* < 0.05), whereas it was non-significant between Aβ-800 pM and Aβ-800 pM+Lin− SC groups. **(C)** In search error, the distance from the hidden platform was significantly reduced in Aβ-1 μM+Lin− SC groups compared to Aβ-1 μM (^*^*p* < 0.05), whereas it was non-significant between Aβ-800 pM and Aβ-800 pM+Lin− SC groups. Data were analyzed using SPSS repetitive measure ANOVA test followed by LSD *post hoc* analysis.
