# Supplementary figures and images for: CD34 and CD117 Stemness of Lineage-Negative Cells Reverses Memory Loss Induced by Amyloid Beta in Mouse Model
Source: Front Behav Neurosci. 2018 Nov 1;12:222. doi: 10.3389/fnbeh.2018.00222 (PMC6222267; doi:10.3389/fnbeh.2018.00222)

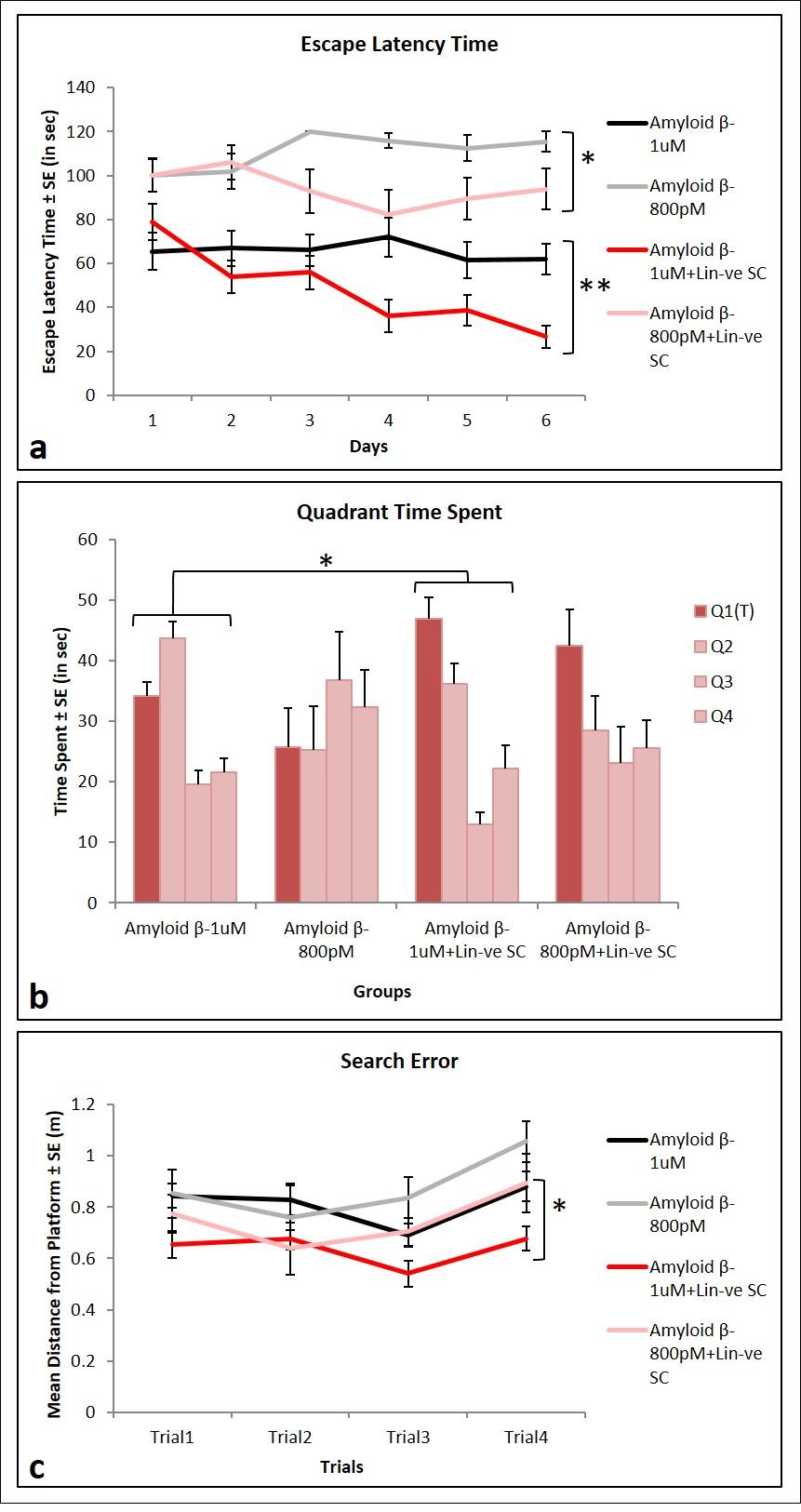

Supplement: Supplementary Figure 1 — MWM analysis shows the difference between 800 pM and 1 μMAβ treated groups (N = 7) with their respective Lin− stem cell transplantation groups. Stem cell-transplanted groups showed significant improvement in cognition in 1 μM of Aβ-injured mice (N = 8) compared to 800 pM dose. (A) In acquisition trials, difference in ELT between Aβ-1 μM and Aβ-1 μM+Lin− SC was significantly higher (**p < 0.001) compared to difference between Aβ-800 pM (N = 5) and Aβ-800 pM+Lin− SC (N = 5; *p < 0.05). (B) In retrieval trials, the quadrant time was significantly improved in Aβ-1 μM+Lin− SC groups compared to Aβ-1 μM (*p < 0.05), whereas it was non-significant between Aβ-800 pM and Aβ-800 pM+Lin− SC groups. (C) In search error, the distance from the hidden platform was significantly reduced in Aβ-1 μM+Lin− SC groups compared to Aβ-1 μM (*p < 0.05), whereas it was non-significant between Aβ-800 pM and Aβ-800 pM+Lin− SC groups. Data were analyzed using SPSS repetitive measure ANOVA test followed by LSD post hoc analysis. [file Image_1.JPEG]
